# Analysis revealed the molecular mechanism of oxidative stress-autophagy-induced liver injury caused by high alkalinity: integrated whole hepatic transcriptome and metabolome

**DOI:** 10.3389/fimmu.2024.1431224

**Published:** 2024-07-08

**Authors:** Xinchi Shang, Longwu Geng, Hai jun Wei, Tianqi Liu, Xinghua Che, Wang Li, Yuhao Liu, Xiao dan Shi, Jianhong Li, Xiaohua Teng, Wei Xu

**Affiliations:** ^1^ Heilongjiang River Fisheries Research Institute, Chinese Academy of Fishery Sciences, Harbin, China; ^2^ Key Laboratory of Cold Water Fish Germplasm Resources and Multiplication and Cultivation of Heilongjiang Province, Harbin, Heilongjiang, China; ^3^ College of Life Science, Northeast Agricultural University, Harbin, China; ^4^ College of Animal Science and Technology, Northeast Agricultural University, Harbin, China

**Keywords:** high alkalinity stress, MiRNA-mRNA network, metabolomics, autophagy, miR-140-5p-ULK2 axis

## Abstract

**Introduction:**

High-alkalinity water is a serious health hazard for fish and can cause oxidative stress and metabolic dysregulation in fish livers. However, the molecular mechanism of liver damage caused by high alkalinity in fish is unclear.

**Methods:**

In this study, 180 carp were randomly divided into a control (C) group and a high-alkalinity (A25) group and were cultured for 56 days. High-alkalinity-induced liver injury was analysed using histopathological, whole-transcriptome, and metabolomic analyses.

**Results:**

Many autophagic bodies and abundant mitochondrial membrane damage were observed in the A25 group. High alkalinity decreased superoxide dismutase (SOD), catalase (CAT), and glutathione peroxidase (GSH-Px) activity and the total antioxidant capacity (T-AOC) and increased the malondialdehyde (MDA) content in liver tissues, causing oxidative stress in the liver. Transcriptome analysis revealed 61 differentially expressed microRNAs (miRNAs) and 4008 differentially expressed mRNAs. Kyoto Encyclopedia of Genes and Genomes (KEGG) enrichment analysis revealed that mammalian target of rapamycin (mTOR), forkhead box O (FoxO), mitogen-activated protein kinase (MAPK), and the autophagy signalling pathway were the molecular mechanisms involved. High alkalinity causes oxidative stress and autophagy and results in autophagic damage in the liver. Bioinformatic predictions indicated that Unc-51 Like Kinase 2 (ULK2) was a potential target gene for miR-140-5p, demonstrating that high alkalinity triggered autophagy through the miR-140-5p–ULK2 axis. Metabolomic analysis revealed that the concentrations of cortisol 21-sulfate and beta-aminopropionitrile were significantly increased, while those of creatine and uracil were significantly decreased.

**Discussion:**

The effects of high alkalinity on oxidative stress and autophagy injury in the liver were analysed using whole-transcriptome miRNA-mRNA networks and metabolomics approaches. Our study provides new insights into liver injury caused by highly alkaline water.

## 1 Introduction

Saline–alkaline water is widely distributed on Earth. Natural soil degradation and overgrazing by pastoralists has resulted in the formation of many saline‒alkali soils (1, 2). Globally, saline–alkaline land covers 0.95 billion hectares, accounting for 1/3 of the total land area. The area of saline–alkaline land in Northeast China is approximately 3.78 × 10^6^ hm2, and most is carbonate saline–alkaline land (3, 4). The saline–alkaline water in the region has characteristics such as low salinity, high alkalinity, and high pH, resulting in low bioavailability (5). The development of fisheries in regions of saline‒alkaline water is important in the utilization of abundant environmental resources. The high alkalinity of saline–alkaline water is highly detrimental to fish, threatening their growth and health and limiting the development of the aquaculture industry (5–7). The water in Qinghai Lake has high alkalinity (carbonate alkalinity of approximately 29 mM, pH 9.1-9.5) (8). Under highly alkaline conditions, blood ammonia levels in crucian carp are significantly increased (1); moreover, ammonia is toxic, and patients with liver dysfunction have higher blood ammonia levels (9). Fish living in saline–alkaline water with high alkalinity can be harmed, slowing their growth and even resulting in their death (5). Alkali stress leads to dysregulation of lipid metabolism in crucian carp and induces cellular apoptosis and an immune response (1). High alkalinity also leads to oxidative stress and inflammation in the spleen in *Luciobarbus capito* (4). However, the mechanism by which high alkalinity leads to liver damage in common carp is unclear.

Integrated multiomics analysis facilitates the systematic study of pathogenesis. Transcriptomic analysis is an efficient approach to simultaneously identify differentially expressed genes (DEGs) and determine their mechanisms of action at the transcriptome level (10). Metabolomic analysis can be used to identify many differentially abundant small molecule metabolites. Integrated transcriptomic and metabolomic analyses revealed that high alkalinity induced apoptosis and immune responses in gill cells of crucian carp and led to dysregulation of glycerophospholipid and arachidonic acid metabolism (1). Therefore, it is important to use transcriptomic and metabolomic analyses to study molecular regulatory mechanisms and pathological states *in vivo*. The liver, a very important organ in animals, is responsible for detoxification. Recent studies have shown that the liver is also a target organ for environmental stressors and that liver damage occurs in common carp under environmental stress (11–13). Unc-51-like kinase 1/2 (*ULK1* and *ULK2*) are mammalian homologues that play crucial regulatory roles in the initiation of autophagy (14). The protein encoded by the *ULK2* gene is a major inducer of autophagy that regulates pyramidal neurons through autophagy and controls the balance between excitation and inhibition in the cerebral cortex (15, 16). A study showed that *ULK2* knockdown inhibits autophagy in porcine testis-supporting cells (17). Short noncoding microRNAs (miRNAs) are non-protein-coding RNA molecules (18). *miR-140-5p* expression is significantly downregulated in patients with osteoarthritis (19). miRNAs can participate in toxin-induced autophagy by targeting autophagy-related genes. Ammonia stress leads to the upregulation of Beclin1, microtubule-associated protein light chain 3 (*LC3*) II and autophagy protein 5 (*ATG5*) mRNA expression and the downregulation of *miR-202-5p*, *LC3-I* and mammalian target of rapamycin (*mTOR*) mRNA expression in chicken heart tissue (20). A recent study revealed that *miR-25-3p* mediates hepatic autophagy in common carp during cadmium poisoning by targeting mRNAs and regulating their expression (11).

Under environmental stress, animals cannot maintain normal physiological functions, resulting in a decreased antioxidant capacity and oxidative stress. Alkali stress results in an increase in the metabolic rate in the context of osmoregulatory energy expenditure and increased production of reactive oxygen radicals, thus affecting the internal antioxidant enzyme system (21). When reactive oxygen species (ROS) accumulate excessively, they induce cellular autophagy by controlling the activity of the autophagy-initiating kinase ULK1 (22). High alkalinity leads to decreases in the levels of the gill antioxidant enzymes superoxide dismutase (SOD) and catalase (CAT) and an increase in the malondialdehyde (MDA) content in crucian carp, disrupting the antioxidant system and leading to oxidative stress (1). Thus, high alkalinity can lead to oxidative stress, and further exploration of the complex mechanism by which high alkalinity leads to autophagy in common carp hepatocytes is needed. This study aimed to reveal the mechanism through which liver damage is induced by high alkalinity in common carp through transcriptomic analysis of miRNAs and mRNAs, metabolomic analysis, tissue and cell morphology analyses, experiments with detection kits, and quantitative real-time PCR (qRT–PCR) analysis.

## 2 Materials and methods

### 2.1 Experimental animals and sampling

Three hundred and sixty healthy common carp juveniles (*Cyprinus carpio*, weighing 20 ± 3 g, body length 12 ± 2 cm) were obtained from the Hulan Experimental Station of Heilongjiang Fisheries Research Institute (Harbin, China). The laboratory adaptation period was seven days. Before use in experiments, all the experimental fish were exposed to fresh dechlorinated water for seven days for environmental adaptation. During the adaptation period, the fish were allowed to eat normally and maintain an active mental state; in addition, during this period, no surface damage occurred, and no fish died. During the experiment, an automatic temperature controller was used to maintain the conditions of the water as follows: a temperature of 23.0 ± 1.0°C, a light/dark cycle of 14/10 hours, a dissolved oxygen concentration of 7.2 ± 0.4 mg/L, an ammonia nitrogen concentration of less than 0.5 mg/L, and a pH of 7.2 ± 0.2. The fish were fed commercial food (Tongwei Co., Ltd, China) twice daily, and half of the water in the tank was changed every day. The corresponding amount of an alkaline buffer was added to the tank, and the alkalinity was measured to maintain the set concentration. This animal study was performed according to the Guidelines for the Feeding and Application of Laboratory Animals of Heilongjiang Fisheries Research Institute, Chinese Academy of Fishery Sciences, and was approved by the Committee on the Ethics of Animal Experiments of Heilongjiang Fisheries Research Institute, Chinese Academy Fishery Sciences (20230728-003).

The alkali stress experiment started on August 1, 2022, and lasted for 56 days. One hundred and eighty healthy common carp (20 ± 3 g) were randomly divided into two groups [the control (C) group (0 mmol/L NaHCO_3_) and the high-alkalinity (A25) group (25.0 ± 0.1 mmol/L NaHCO_3_)], with three glass tanks (each containing 180 L of water) per group and 30 fish per tank. The concentration of NaHCO_3_ used for the A25 group was based on two papers: one that reported that an alkalinity of 150-300 mg/L is high (23), and another providing data from regions in China with highly saline–alkaline water in aquatic ecosystems (3). The alkalinity of water in northern China is generally 15-20 mmol/L, and fish in this region grow slowly. Therefore, in this study, a high alkalinity (25 mmol/L) was chosen to explore the effects of high alkalinity on fish. This study provides a reference for aquaculture under conditions of high alkalinity. On the 56^th^ day of the experiment, six fish were randomly selected from each tank in both the C group and the A25 group. The fish were euthanized by administration of MS-222 (100 mg/L, Sigma, USA), and blood and liver tissues were collected. The collected liver tissues were subjected to microstructural observation, ultrastructural observation, analysis with detection kits, whole-transcriptome analysis, and metabolomic analysis.

### 2.2 Microstructural and ultrastructural observation

The method used for the preparation of microstructural sections of the livers of twelve fish was described by Cui et al. (12). In brief, liver sections were fixed with 10% paraformaldehyde. The sections were dehydrated with ethanol and immersed in xylene. Paraffin-embedded liver samples were sliced into sections using a pathological sectioning instrument. The sections were stained with haematoxylin and eosin (H&E). ZYscanner scanning software was used to acquire high-resolution digital images of the sections in the full field of view.

Ultrastructural sections of the livers from twelve fish were prepared. In brief, fixed liver tissue blocks were removed from the 2.5% glutaraldehyde solution, washed three times with 0.1 M phosphate buffer, soaked in 1% osmium tetroxide for 3 hours, and dehydrated in ethanol and pure acetone. The tissue blocks were then embedded in epoxy resin, cut into ultrathin sections of 50-60 nm and stained with uranyl acetate and lead citrate. The ultrastructure was observed using a transmission electron microscope (H7650, Hitachi, Tokyo, Japan).

### 2.3 Detection kits

Blood ammonia and oxidative stress-related indexes were evaluated with assay kits. Serum was separated from blood samples collected from twelve fish after 12 hours of incubation in a refrigerator at 4°C, and blood ammonia levels were subsequently measured according to the instructions of the blood ammonia assay kit (Nanjing Jiancheng Institute of Biological Engineering, Nanjing, Jiangsu, China). The obtained 0.1 g liver samples were homogenized in 0.9 mL of 0.86% saline solution using a motor-driven tissue grinder (D-160, Beijing Dalgng, Xingchuang Experimental Instrument Co., Ltd, Beijing, China). The homogenate was centrifuged at 4 °and 1000 rpm for 15 min. The supernatant was collected for the determination of the total antioxidant capacity (T-AOC), the MDA content, and SOD, CAT, and glutathione peroxidase (GSH-Px) activity according to the manufacturer’s instructions.

### 2.4 Transcriptome sequencing

Transcriptome sequencing analysis of the livers from six fish was carried out by Lianchuan Biological Co., Ltd (Hangzhou, China). The main steps were as follows. Total RNA from liver tissues was extracted with TRIzol reagent following the manufacturer’s instructions (Takara Co. Ltd, Beijing, China). The total RNA samples obtained were subjected to miRNA sequencing (miRNA-seq) and mRNA sequencing (mRNA-seq).

#### 2.4.1 miRNA-seq

RNA samples were obtained using the TruSeq™ Small RNA Sample Preparation Kit, sRNA libraries were constructed, and single-end sequencing (1 × 50 bp) was performed. The Illumina raw reads were evaluated using FastQC to obtain the clean Q30 reads. Clean reads with a length distribution of 18 to 25 nt were counted. Known conserved miRNAs were identified, and novel miRNAs were predicted using ACGT101-miR data analysis software (LC Sciences, Houston, TX). miRNA precursor secondary structures were predicted using RNAfold. miRNA expression was normalized to each library size on a transcripts per million basis. Differential expression of miRNAs was analysed using Student’s t test. The significance threshold was set at 0.05. Differentially expressed carp miRNAs (DEMs) were functionally annotated via Gene Ontology (GO) enrichment analysis and subjected to GO and Kyoto Encyclopedia of Genes and Genomes (KEGG) enrichment analyses.

#### 2.4.2 Analysis of mRNA-seq data

Sequencing was performed on the Illumina NovaSeq™ 6000 platform. Clean reads were obtained using Cutadapt software and were mapped to the genome using HISAT2 software. The NCBI *Cyprinus carpio* (carp) RefSeq GCF_000951615.1 assembly was used as the reference genome. After generation of the final transcripts, mRNA expression levels were calculated using FPKM. Differential gene expression analysis was performed between the two groups using DESeq2 software. Genes with a false discovery rate (FDR) of less than 0.05 and an absolute fold change in expression of ≥ 2 were considered DEGs. All DEGs were mapped to GO terms in the GO database. Genomic enrichment analyses were performed using GSEA software and MSigDB to determine whether a set of genes associated with a particular GO term and KEGG pathway differed significantly between the two groups. GO terms and KEGG pathways with Pval<0.05 were considered differentially enriched between the two groups.

### 2.5 miRNA‒mRNA network analysis

The prediction and analysis of target genes were performed by Lianchuan Biotechnology Co., Ltd (Hangzhou, China). Target genes were predicted using TargetScan and miRanda. The miRNA expression profile and mRNA expression profile were integrated to identify the key miRNAs and their corresponding target genes. A miRNA‒mRNA regulatory network was constructed based on the regulatory relationships between miRNAs and mRNAs identified in this study.

### 2.6 Analysis of miRNA and mRNA transcription

qRT‒PCR was used to measure the transcript levels of miRNAs and mRNAs. Stored liver samples from six fish were removed from the refrigerator, and total RNA was extracted with TRIzol reagent (TaKaRa, Dalian, China). Five DEMs and 10 DEGs (3 autophagy-related genes, 3 antioxidant-related genes, and 4 randomly selected genes) were selected and analysed via qRT‒PCR to measure their transcript levels in carp liver. The PCR sequences of the primers used in this study are listed in **Table 1**. Relative gene expression was calculated by the 2^-ΔΔCT^ method.

**Table 1:** Primers used in this study.

Gene		Primer sequence (5′ - 3′)
*β-actin*	Forward Reverse	TGAAGATCCTGACCGAGCGT GGAAGAAGAGGCAGCGGTTC
*ccr-miR-140-5p*	Forward Reverse	CAGTGGTTTTACCCTATGGTAG Provided by Tiangen Biotech Co. Ltd
*ccr-mir-26a-p3*	Forward Reverse	CCTATTCATGATTACTTGCACT Provided by Tiangen Biotech Co. Ltd
*PC-5p-10768_71*	Forward Reverse	ACTCAGTACTCAGTGTAGGGTC Provided by Tiangen Biotech Co. Ltd
*ccr-mir-122-p5_1ss4TC*	Forward Reverse	CTGCCGTCCTCCTGAGCTG Provided by Tiangen Biotech Co. Ltd
*dre-miR-92a-3p*	Forward Reverse	TATTGCACTTGTCCCGGCCTGT Provided by Tiangen Biotech Co. Ltd
*ULK2*	Forward Reverse	TCCCCATTCATCCACTGTGC AGGCTTTGTAGGCTCACGAC
*Mapk1*	Forward Reverse	CTCAGGCGTTGGTCTGGATT ATCAGAGGACTGCGAGAGGT
*Nrf1*	Forward Reverse	GTGATGGAAGACCACACGGT AGCATCATCATCAGGCGAGG
*Akt1s1*	Forward Reverse	GTGTGGGGCTACAGGAACAA CCGTGTTAAGGCGAGGTCTT
*Keap1*	Forward Reverse	GCTGCACAAGCCCACTAAAC ATGACACAGGCTGCTAACCC
*Atg101*	Forward Reverse	AACCTGGCCAACGAACAAGA CACTGACGTGCCCAGAGAAT
*LOC109106952*	Forward Reverse	CAGGTGCAGGAAAAACCACG AGATGTCTGACTCTCGGCCT
*LOC109103689*	Forward Reverse	ATGAAGTCCAGTCAGTGCGG ACGCGCAAGTACCACCATTA
*LOC109103224*	Forward Reverse	CCTCCTGCAAGTCAACTCGT GATAACGAGGTCGGCAGAGG
*LOC109074642*	Forward Reverse	TGGATCAGCCGAACTCTCCT ACTGAGGCATAACCGTGCTC
*ULK1*	Forward Reverse	CGTGACCTGAAGCCCCAAAA CGAAGTCCATGCGCTCCCTAT
*mTOR*	Forward Reverse	CGGTGCTGGTTTTGCGAGAG TGGTGAAGGGCGTGATGTGG
*LC3*	Forward Reverse	GCCTTCGTTGGCTATGTTCT GGTTTGATGTGGGGTGGTGT
*ATG5*	Forward Reverse	GTCATGAAGGCCGAAGACGTG ATTCTAAAGGGAATATAGCGGAAGC

### 2.7 Untargeted hepatic metabolome analysis

Metabolites were extracted from the livers of twelve fish using the 50% methanol buffer precipitation method, and quality control (QC) samples were prepared simultaneously. The extracted samples were subjected to random on-board sequencing analysis, and the QC samples were injected before, during and after the test samples for repeated assessment of the experimental technique. The metabolites eluted from the column were detected by HPLC-HRMS. The samples were analysed by mass spectrometry in positive and negative ion modes. Data were preprocessed using XCMS software. metaX software was used for data analysis.

Substances were quantified and screened for differential abundance using metaX software. Specifically, metaX software was used to match the primary m/z corresponding to each product with the metabolites in the PlantCyc, KEGG, and HMDB databases for primary identification; moreover, an in-house library was used to identify the metabolites in the secondary mass spectra of the products. The metaX software was used for univariate and multivariate analyses of the metabolomic data to identify differentially abundant metabolites (DMs) between the groups. Differential abundance was assumed when three conditions were met simultaneously: (1) a multiplicity of substances ≥ 2 or a ratio ≤ 1/2; (2) a Benjamini–Hochberg (BH)-adjusted q value of in the Wilcoxon rank–sum test; and (3) A variable projected importance (VIP) value ≥1 in the orthogonal partial least squares discriminant analysis (OPLS-DA) model. The KEGG identifier of each DM was determined, the KEGG homepage was accessed, the analysis tool KEGG Mapper was used, the KEGG identifier of each differentially abundant metabolite was entered, and the metabolic network was mapped in conjunction with each of the connecting pathways.

### 2.8 Statistical analysis

Statistical analysis was performed using SPSS 20.0 software, and the data are expressed as the mean ± SD values. The significance of differences between the two groups were evaluated by two-tailed unpaired Student’s t test (parametric). For all the statistical tests, *P* < 0.05 indicated statistical significance.

## 3 Results

### 3.1 Liver microstructure and ultrastructure

**Figure 1:**
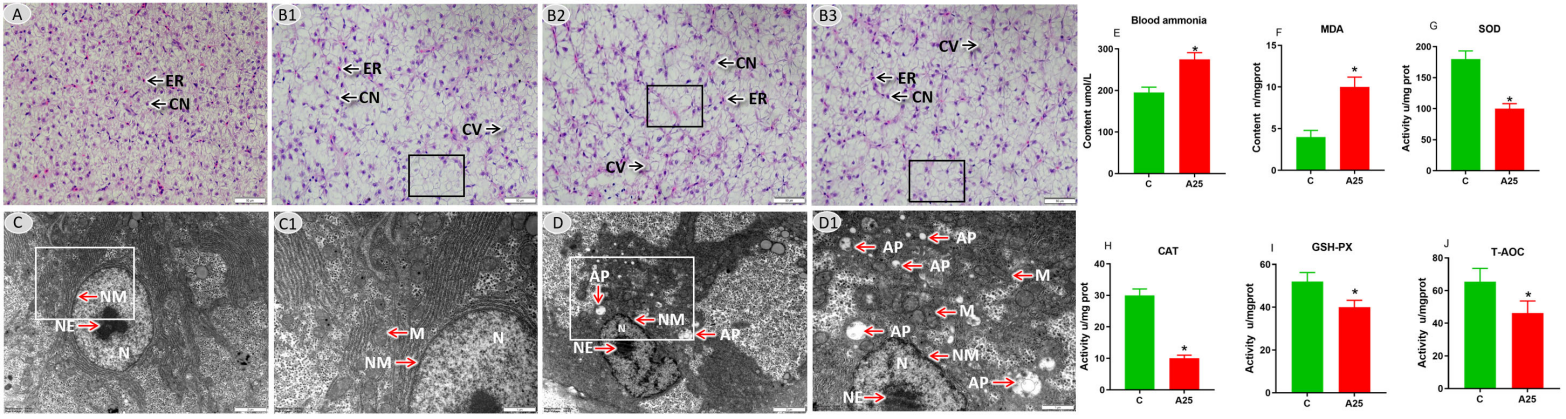
Representative images of liver tissues. **(A)** Microstructure in the C group; scale bars = 50 µm (40 ×). **(B1–B3)** Microstructure in the A25 group; scale bars = 50 µm (40 ×). Nuclei of hepatocytes (CNs) and erythrocytes (ERs), hepatic cell vacuolization (CVs), and lamellar vacuolation of cells (black boxes). **(C)** Ultrastructure in the C group; scale bar = 2 µm (10,000 ×). **(C1)** Magnified image of the area enclosed in the rectangle in C; scale bar = 1 µm (20,000 ×). **(D)** Ultrastructure in the A25 group; scale bar = 2 µm (10,000 ×). **(D1)** Magnified image of the area enclosed in the rectangle in b1; scale bar = 1 µm (20,000 ×). Mitochondrion (M), autophagosome (AP), nucleoplasm (N), nucleolus (NE), nuclear membrane (NM). Blood ammonia and hepatic oxidative stress-related indexes **(E–J)**. **(E)** Blood ammonia content, **(F)** MDA content, **(G)** SOD activity, **(H)** CAT activity, **(I)** GSH-PX activity, **(J)** T-AOC activity. The data in this figure are presented as the mean ± SD of three parallel measurements. * indicates a significant difference (*P* < 0.05) between the C group and the A25 group (n = 6 fish per group).

Pathological changes in the liver of common carp under high-alkalinity conditions were investigated. The microstructure is shown in **Figure 1A**. The C group showed a healthy tissue structure with features such as a regularly arranged hepatic parenchyma. The A25 group (**Figure 1B**) showed pathological changes in liver tissues, such as cell enlargement and nuclear pyknosis (CN), cellular vacuolization (CV), and lamellar vacuolization of hepatocytes (black square). Via electron microscopy, as shown in **Figures 1C, D**, the C group showed an intact cellular structure (**Figure 1C**). However, the A25 group (**Figure 1D**) showed pathological changes in hepatocytes, such as mitochondrial membrane damage (M), many autophagic bodies (APs), swelling and rupture of the endoplasmic reticulum (RER), and nuclear membrane atrophy (NM).

### 3.2 Effects of exposure to high alkalinity on blood ammonia and hepatic oxidative stress-related indices

We explored the effects of exposure to high alkalinity for 60 days on blood ammonia and hepatic oxidative stress-related indices (**Figure 1**) and found that the contents of blood ammonia (**Figure 1E**) and MDA (**Figure 1F**) were significantly increased (*P* < 0.05) in the A25 group compared with the control group. In contrast (**Figures 1G–J**), the activities of SOD, CAT, GSH-Px, and T-AOC were significantly decreased (*P* < 0.05) in the A25 group.

### 3.3 mRNA-seq analysis results

**Figure 2:**
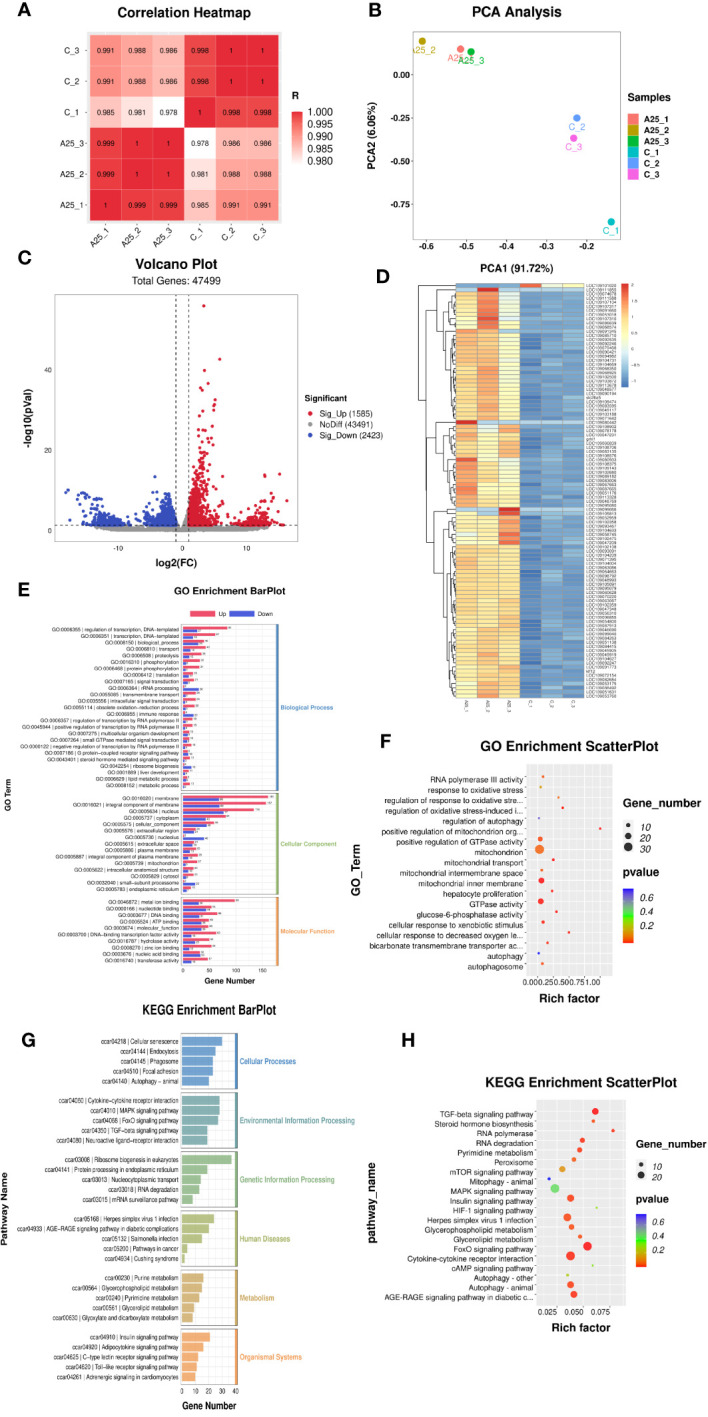
mRNA-seq results. Correlation analysis of gene expression data in the samples **(A)**. Principal component analysis (PCA) plot **(B)**. Volcano plot **(C)** and heatmap **(D)** of the DEGs (*P* < 0.05). Enrichment bar chart for GO terms **(E)** and enrichment factor scatter plot **(F)** for GO terms. Enrichment bar chart for KEGG pathways **(G)** and enrichment factor scatter plot for KEGG pathways **(H)**.

Pearson correlation analysis revealed that the Pearson correlation coefficients were greater than 0.9 for all six samples, as shown in **Figure 2A**, indicating that all six samples were similar across individuals from the same population. The principal components identified by PCA explained 97.78% of the variability among the six samples. This finding indicated that the experimental model that we designed was accurate and that our transcriptome data were reliable (**Figure 2B**). The levels of 4008 genes were significantly altered by alkali treatment (A25 group), among which 1585 were significantly upregulated and 2423 were significantly downregulated. The volcano plot visualizing differential gene expression is shown in **Figure 2C**. The differential gene expression heatmap provides a clear visualization of the gene expression patterns. The numbers of up- and downregulated genes are shown in **Figure 2D**.

Significantly enriched (*P* < 0.05) GO terms were identified using the GO enrichment analysis method (OmicStudioKits). The BP, CC, and MF terms were sorted according to the number of differentially annotated genes (S gene number) in descending order, and the top 25 BP terms, top 15 CC terms, and top 10 MF terms were selected for mapping and presentation. The enrichment bar chart is shown in **Figure 2E**. The results of ggplot2 analysis of significant enrichment (*P <*0.05) as determined by GO analysis are presented as bubble plots. The enrichment factor scatter plot is shown in **Figure 2F**. GO enrichment analysis revealed that terms related to the regulation of the oxidative stress-induced intrinsic apoptotic signalling pathway and mitochondrial transport were significantly enriched in the BP category. Autophagosome, mitochondrial inner membrane, and mitochondrial intermembrane space were the significantly enriched terms in the CC category. Glucose-6-phosphatase activity, GTPase activity, and bicarbonate transmembrane transporter activity were the significantly enriched terms in the MF category.

The KEGG database was used to annotate the pathways associated enriched in the DEGs. The KEGG pathways enriched in these genes could be divided into six categories. The enrichment bar chart is shown in **Figure 2G**. The most significantly enriched pathways in these six categories were as follows: Autophagy-animal, Focal adhesion, and Phagosomes were the significantly enriched terms in the Cellular Processes category; TGF-beta signalling pathway, FoxO signalling pathway, and MAPK signalling pathway were the significantly enriched terms in the Environmental Information Processing category; mRNA surveillance pathway was the enriched term in the Genetic Information Processing category; Salmonella infection and Herpes simplex virus 1 infection were the significantly enriched terms in the Human Diseases category; Glycerophospholipid metabolism was the significantly enriched term in the Metabolism category; and Adrenergic signalling in cardiomyocytes and Toll-like receptor signalling pathway were the significantly enriched terms in the Organismal Systems category. The results of ggplot2 analysis of significant enrichment as determined by KEGG analysis are presented as bubble plots. The enrichment factor scatter plot is shown in **Figure 2H**. Our analysis identified the 20 most significantly enriched KEGG pathways. High alkalinity was found to be closely related to hepatic oxidative stress- and autophagy-related signalling pathways. The significantly enriched pathways included the FOXO signalling pathway, autophagy-animal pathway, peroxisome pathway, and TGF-beta signalling pathway.

### 3.4 miRNA-seq analysis results

**Figure 3:**
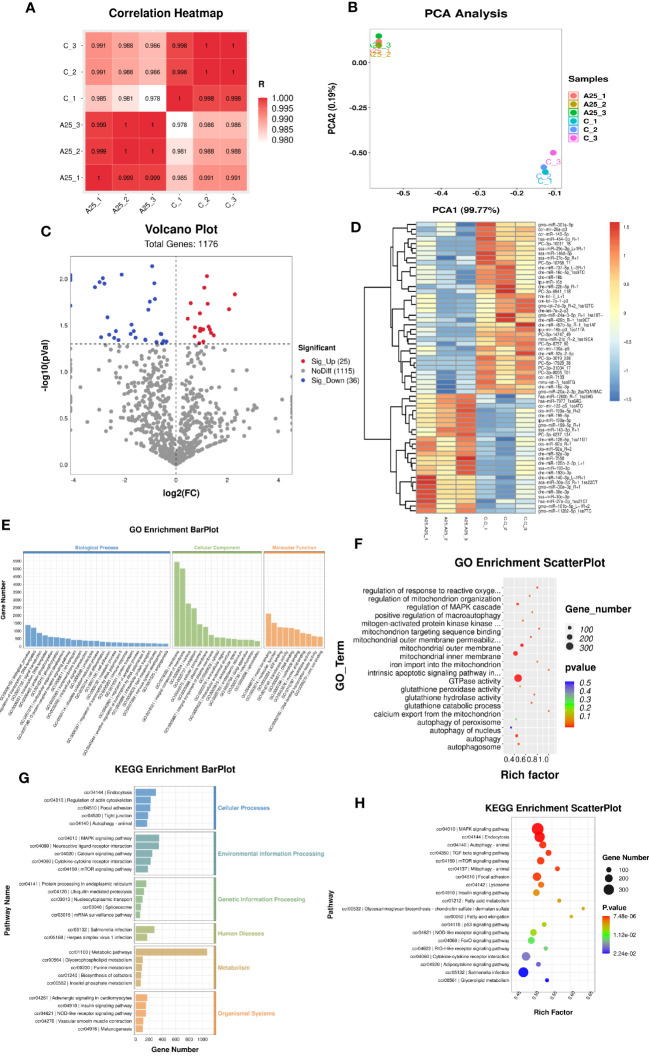
miRNA-seq analysis results. Correlation analysis of gene expression data in the samples **(A)**. Principal component analysis (PCA) plot **(B)**. Volcano plot **(C)** and heatmap **(D)** of the DEGs (*P* < 0.05). Enrichment bar chart for GO terms **(E)** and enrichment factor scatter plot for GO terms **(F)**. Enrichment bar chart for KEGG pathways **(G)** and enrichment factor scatter plot for KEGG pathways **(H)**.

To explore the mechanisms by which miRNAs are involved in alkali stress in the liver, miRNA-seq was carried out. Pearson correlation analysis revealed that all six samples were similar across individuals from the same population (**Figure 3A**), and PC1 and PC2 identified by PCA explained 99.77% and 0.19% of the variability among the six samples, respectively. This finding indicated that the experimental model that we designed was accurate and that our transcriptome data were reliable (**Figure 3B**). Furthermore, we identified 61 DEMs (**Figure 3C**). The heatmap shows the expression levels of the 61 DEMs, and the miRNAs with consistent expression patterns were clustered together (**Figure 3D**). The potential target genes of the miRNAs were explored by GO and KEGG enrichment analyses.

Significantly enriched (*P* < 0.05) GO terms were identified using the GO enrichment analysis method (OmicStudioKits). Among all the selected DEM target genes, we classified the GO terms enriched in these genes into three categories according to BP, CC, and MF and explored their important biological functions. The top 25 BP terms, top 15 CC terms, and top 10 MF terms were selected for mapping and presentation. The enrichment bar chart is shown in **Figure 3E**. The results of ggplot2 analysis of significant enrichment (*P*<0.05) as determined by GO analysis are presented as bubble plots. The enrichment factor scatter plot is shown in **Figure 3F**. GO enrichment analysis revealed that the BP terms autophagy, intrinsic apoptotic signalling pathway in response to oxidative stress, regulation of response to oxidative stress, and regulation of MAPK cascade were significantly enriched. Autophagosome, mitochondrial inner membrane, and mitochondrial outer membrane were the significantly enriched CC terms. The MF terms protein kinase inhibitor activity, GTPase activity, mitochondrion targeting sequence binding, and glutathione peroxidase activity were significantly enriched.

KEGG analysis was performed to identify the pathways significantly enriched in the target genes. The KEGG pathways enriched in the target genes of the DEMs were classified into six categories. The enrichment bar chart is shown in **Figure 3G**. The most significantly enriched pathways in these six categories were as follows: autophagy-animal was the significantly enriched pathway in the Cellular Processes category; MAPK signalling pathway and mTOR signalling pathway were the significantly enriched pathways in the Environmental Information Processing category; protein processing in the endoplasmic reticulum was the significantly enriched pathway in the Genetic Information Processing category; Salmonella infection was the significantly enriched pathway in the Human Diseases category; metabolic pathways was the significantly enriched pathway in the Metabolism category; and vascular smooth muscle contraction was the significantly enriched pathway in the Organismal Systems category. The results of ggplot2 analysis of significant enrichment as determined by KEGG analysis are presented as bubble plots. The enrichment factor scatter plot is shown in **Figure 3H**. Our results identified the 20 most significantly enriched KEGG pathways. High alkalinity was found to be closely related to hepatic oxidative stress- and autophagy-related signalling pathways. The top five significantly enriched pathways were the MAPK signalling pathway, endocytosis pathway, autophagy-animal pathway, TGF-beta signalling pathway, and mTOR signalling pathway.

### 3.5 Integrated miRNA and mRNA analysis

**Figure 4:**
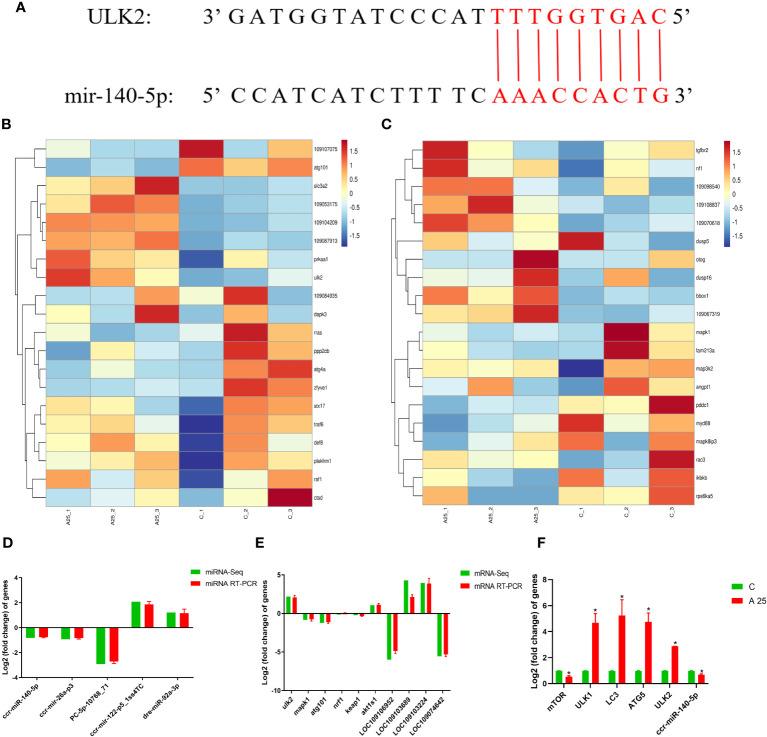
Integrated analysis or interactions between miRNAs and their target mRNAs. Prediction results for base complementarity **(A)**; Top 20 target mRNAs related to autophagy **(B)** and oxidative stress **(C)** in GO terms and KEGG pathways; Verification of the miRNA-seq **(D)** and mRNA-seq **(E)** results using qRT‒PCR; Gene expression in liver tissues of carp in the C and A25 groups **(F)**. Statistical significance was determined by Student’s t test. *P < 0.05.

The identified miRNAs and mRNAs were analysed, and differentially expressed target mRNAs were obtained based on the regulatory relationships between the mRNAs and miRNAs. The 25 upregulated DEMs were associated with 6861 downregulated DEGs, and the 36 downregulated DEMs were associated with 5656 upregulated DEGs (**Supplementary Table S1**). The predicted target gene for *PC-5p-17929_38*, *ccr-miR-140-5p*, *dre-miR-140-3p_L-1R+1*, *dre-miR-193b-3p*, *dre-miR-22b-5p_R-1*, and *ssa-miR-193-3p* was *ULK2*. The predicted target gene for *gmo-miR-101b-5p_L-1R+2* was *atg101*. The predicted target gene for *gmo-miR-20a-2-3p_2ss7GA19AC* was *nrf1*. The predicted target gene for *PC-3p-31034_17* was *keap1*. Analysis of miRNA and mRNA sequencing data revealed that *miR-140-5p* was downregulated and that *ULK2* was upregulated (**Table 2**). Via a bioinformatic prediction approach, we found that *ccr-miR-140-5p* and *ULK2* have 15 contiguous complementary base pairs (**Figure 4A**). The 20 mRNAs (**Figure 4B**) most strongly associated with autophagy were related to 3 GO terms (autophagosome, regulation of autophagy and autophagy) and 2 KEGG pathways (mTOR signalling pathway and autophagy-animal). The 20 mRNAs (**Figure 4C**) most strongly associated with oxidative stress were related to 2 GO terms (oxidation‒reduction process and oxidoreductase activity) and 2 KEGG pathways (MAPK signalling pathway and TGF-beta signalling pathway).

**Table 2:** Transcriptome sequencing results for *miR-140-5p* and *ULK2*.

Gene	C	A25	Log_2_FC
*miR-140-5p*	2801	1586	-0.82
*ULK2*	1.36	6.24	2.19

### 3.6 Verification of miRNA-seq and mRNA-seq results using qRT‒PCR

In this study, the levels of 5 miRNAs and 10 mRNAs were measured using qRT‒PCR to verify the reliability of the miRNA-seq and mRNA-seq data (**Figures 4D, E**). The expression of the miRNAs *ccr-miR-140-5p, ccr-mir-26a-p3* and *PC-5p-10768_71* was decreased. The expression of the miRNAs *ccr-mir-122-p5_1ss4TC* and *dre-miR-92a-3p* was increased. *ULK2 nrf1* and *akt1s1* were upregulated mRNAs. *Keap1*, *mapk1* and *atg101* were downregulated mRNAs. The qRT‒PCR and RNA-seq results indicated that the transcriptome sequencing data were reliable. In addition, the qRT‒PCR results (**Figure 4F**) showed that the expression of the mTOR mRNA and *ccr-miR-140-5p* was significantly downregulated and that the expression of *ULK1*, *LC3*, *ATG5* and *ULK2* mRNA was significantly upregulated in the A25 group compared to the C group.

### 3.7 Evidence from metabolomic analysis

**Figure 5:**
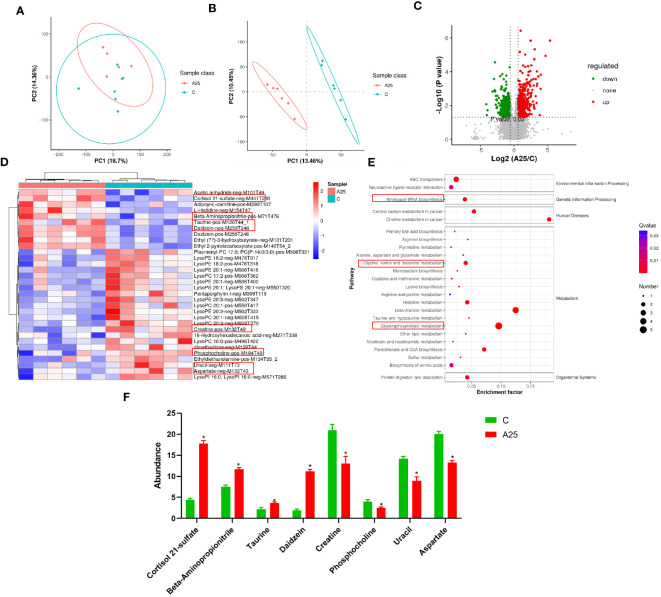
Analysis of metabolomic data quality and metabolic pathways enriched in the significantly differentially abundant metabolites. **(A)** PCA score plot of the samples acquired; **(B)** OPLS-DA score plot; Volcano plot **(C)** and cluster heatmap **(D)** of the differentially abundant metabolites; **(E)** KEGG pathway enrichment analysis results; **(F)** Selected potential metabolite biomarkers. Statistical significance was determined by Student’s t test. The data in this figure are presented as the mean ± SD of three parallel measurements (n = 6 fish per group). *P < 0.05.

Differences in metabolite abundances in carp livers were evaluated by PCA and OPLS-DA multivariate analysis. The PCA score plot is shown in **Figure 5A**. OPLS-DA is a supervised procedure for discriminant analysis of variance that maximizes the differences between groups. The QC samples showed strong clustering in a smaller area between the groups. This pattern indicates the significant separation between the C and AS25 groups (**Figure 5B**). We were able to identify the significant DMs. As shown in the volcano plot (**Figure 5C**), 1015 significant DMs were identified, among which 519 (277 upregulated and 242 downregulated) were identified in positive ion mode, and 496 (255 upregulated and 241 downregulated) were identified in negative ion mode (**Figure 5D**). In this study, after alkali treatment, the concentrations of cortisol 21-sulfate, taurine, daidzein, and beta- aminopropionitrile were significantly increased. However, alkali treatment resulted in significant decreases in the concentrations of creatine, uracil, and aspartate (**Figure 5F**). **Supplementary Table S2** shows the details of the metabolites.

KEGG analysis was performed to identify the pathways significantly enriched in the DMs. We classified the KEGG pathways enriched in all the selected DMs into five categories. The most significantly enriched pathways in these five categories were as follows: neuroactive ligand‒receptor interaction and ABC transporters was the significantly enriched pathway in the Environmental Information Processing category; aminoacyl-tRNA biosynthesis was the enriched pathway in the Genetic Information Processing category; choline metabolism in cancer and central carbon metabolism in cancer were the significantly enriched pathways in the Human Diseases category; glycerophospholipid metabolism, taurine and hypotaurine metabolism, beta-alanine metabolism, pyrimidine metabolism, and histidine metabolism were the significantly enriched pathways in the Metabolism category; and protein digestion and absorption was the significantly enriched pathway in the Organismal Systems category. The results of ggplot2 analysis of significant enrichment as determined by KEGG analysis are presented as bubble plots (**Figure 5E**).

### 3.8 Integrated transcriptomic and metabolomic analysis

**Figure 6:**
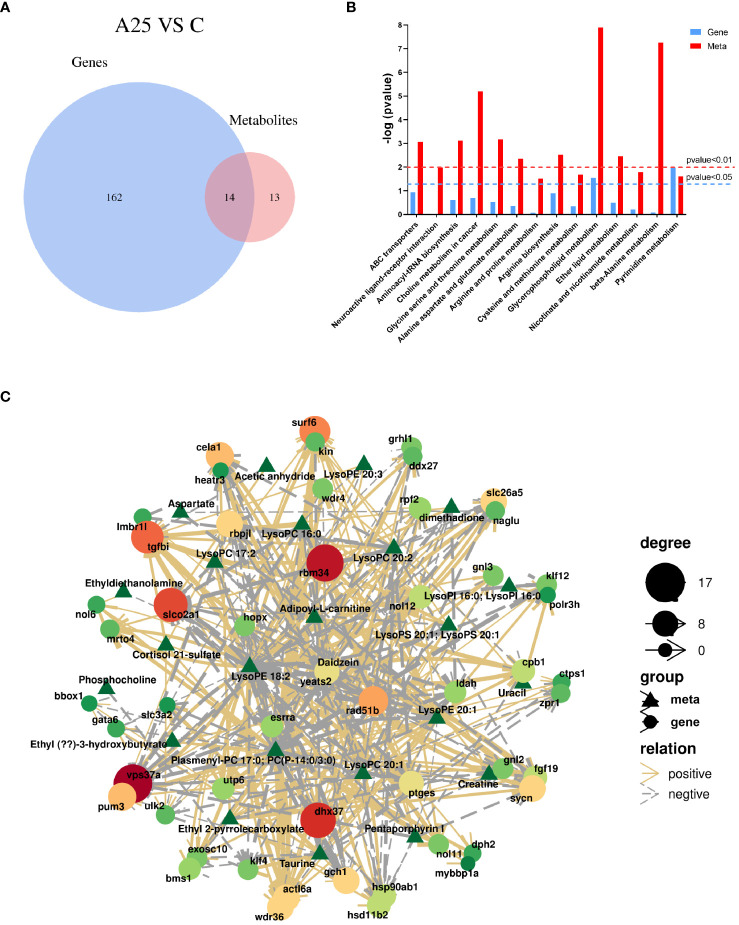
Integrated transcriptomic and metabolomic analysis. **(A)** Venn diagram of the enriched KEGG pathways based on the transcriptomic and metabolomic data. **(B)** Significance analysis of the differentially enriched pathways. **(C)** Analysis of the associations between differentially abundant metabolites and differentially expressed genes.

We integrated the differentially enriched KEGG pathways with the DEGs and DMs from the transcriptomic and metabolomic data. In this study, 176 pathways were enriched based on the transcriptomic data, and 27 metabolic pathways were enriched based on the metabolomic data, with 14 metabolic pathways enriched based on both sets of data (**Figure 6A**). We analysed the 14 coenriched pathways for significant differences and found that metabolic pathways were significantly enriched (*P*<0.05). Glycerophospholipid metabolism and pyrimidine metabolism were the pathways that were significantly coenriched based on the transcriptomic and metabolomic data (**Figure 6B**). Furthermore, we correlated the DMs with the DEGs. The results revealed a positive correlation between the expression of the *ULK2* gene and the abundance of the metabolite cortisol 21-sulfate (**Figure 6C**).

## 4 Discussion

**Figure 7:**
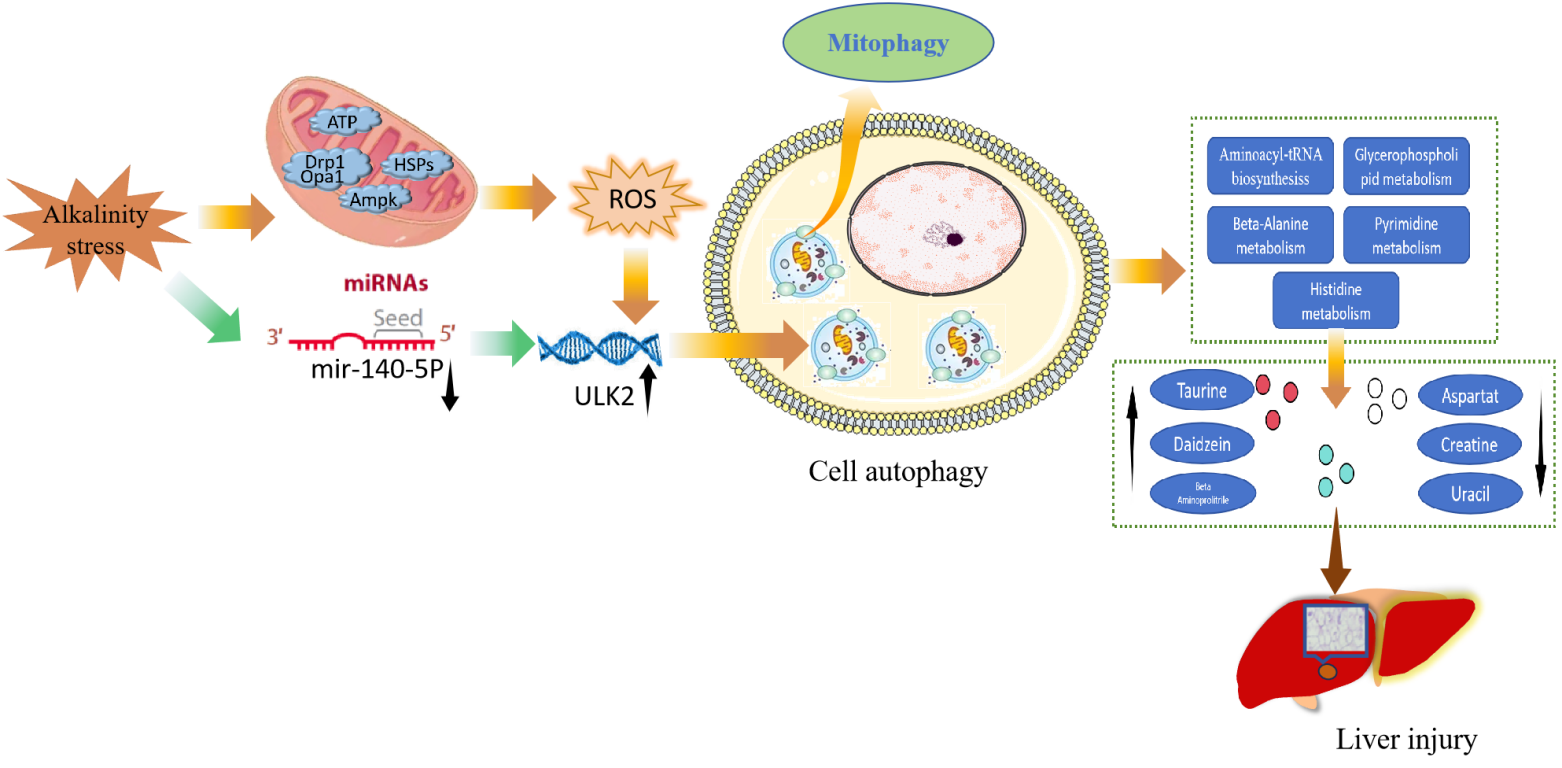
Potential mechanism through which oxidative stress-mediated autophagy-induced metabolic dysregulation leads to liver injury in carp after exposure to high alkalinity.

Saline–alkaline waters are widely distributed worldwide, and most freshwater fish cannot survive in these waters. The rapid development of fisheries production in saline–alkaline waters can be promoted by studying the molecular mechanisms through which alkali stress affects fish. In the present study, high alkalinity triggered hepatic oxidative stress- and autophagy-related pathways and resulted in metabolic dysregulation that causes liver injury in carp. A schematic representation of the potential mechanisms through which high alkalinity induces liver damage is shown in **Figure 7**. Environmental stress leads to liver injury, harmful substances in the environment can induce oxidative stress in animals, and ROS production is the main cause of liver injury (24). ROS induce cellular autophagy by controlling the activity of the autophagy-initiating kinase ULK (22). Autophagy is generally an early response that promotes cell survival, and excessive autophagy can lead to tissue damage. mTOR is the central hub of autophagy regulation and is regulated by different upstream signalling pathways to modulate autophagy (25). The mitogen-activated protein kinase (MAPK) signalling pathway can mediate oxidative stress, inflammation and apoptosis in animal cells (26). Excessive autophagy in mitochondria induces cell death, and PINK1 accumulates in the outer mitochondrial membrane, leading to ubiquitin phosphorylation and PRKN recruitment, facilitating further recruitment of the autophagy machinery to initiate autophagosome formation and mitochondrial autophagy (27). GTPase family proteins are key components of the mechanism mediating mitochondrial fusion and division, and autophagy is induced by GTP hydrolysis (28). In the present study, we found that high alkalinity induced oxidative stress and autophagy in carp livers through the mTOR, forkhead box O (FOXO), MAPK and autophagy signalling pathways. Furthermore, GO enrichment analysis revealed that the target genes of miRNAs are involved in autophagy induced by exposure to high alkalinity.

miRNAs participate in many biological processes in animals, and harmful substances in the environment can alter miRNA expression (29, 30). miRNAs are key regulatory factors for posttranscriptional gene silencing (31). Autophagy is also a mechanism for responding to injury caused by exposure to harmful environmental pollutants, and miRNAs participate in the toxin-induced autophagy mechanism by targeting autophagy-related genes. Ammonia stress leads to the upregulation of the mRNA expression of *Beclin1*, *LC3-II*, and *ATG5* in chicken hearts, and *miR-202-5p* triggers autophagy in chicken hearts by targeting PTEN (20). ULK1/2 play a key regulatory role in the initiation of autophagy (14, 32), and *ULK2* is an autophagy inducer (15). A study revealed that *miR-26a* inhibited autophagy in porcine testis-supporting cells by targeting and regulating *ULK2*, and *ULK2* knockdown inhibited autophagy in porcine testis-supporting cells (17). MAPK1 is activated under mitochondrial autophagy-inducing conditions, such as starvation and hypoxia, and when the upstream kinase *mapk1 was knocked down* in cells, mitochondrial autophagy was severely inhibited (33). A study revealed that the expression of human papillomavirus type 16 early protein E7 decreased the *dusp5* level, which in turn led to activation of MAPK/ERK signalling and induced canonical autophagy through the regulation of mTOR and MAPK in normal human epidermal keratinocytes (34, 35). In the present study, transcriptomic analysis revealed that *miR-140-5p* was downregulated and *ULK2* was upregulated, and the bioinformatic predictions indicated that the potential target gene of *miR-140-5p* was *ULK2*. In addition, our morphological studies revealed that high alkalinity caused autophagic and mitochondrial damage to the livers of common carp. Ampk-ULK1 signalling participates in long-term exercise-induced mitochondrial autophagy in skeletal muscle (36). Under hypoxic stress, ULK1 translocates to dysfunctional mitochondria and participates in mitochondrial autophagy (37). Ampk-ULK2 signalling can also mediate mitochondrial autophagy, and ULK2 knockdown can alleviate mitochondrial autophagy induced by β-amyloid protein 1-42 in mouse neural cells (38). These observations provide further evidence that high alkalinity triggers mitochondrial autophagy in common carp hepatocytes via the *miR-140-5p/ULK2* axis.

To more effectively adapt to extreme environmental changes, organisms must regulate their metabolic processes to satisfy their nutrient requirements. In our study, autophagy was found to lead to metabolic dysregulation in the liver. Research has shown that threonine, glycine, and serine can disrupt the mitochondrial membrane potential, mitochondrial membrane permeability, and mitochondrial synthetic cleavage processes and promote cellular autophagy (39). High cortisol levels activate the hypothalamic‒pituitary‒adrenal axis to promote adrenal secretion of glucocorticoids, which promotes cellular autophagy and contributes to psychiatric disorders and cardiovascular and autoimmune diseases (40). Histidine plays a vital role in the maintenance of cellular homeostasis; it can prevent cellular ROS generation, GSH oxidation, lipid peroxidation, and protein carbonylation, and disrupted histidine metabolism can exacerbate myocardial apoptosis by inducing excessive autophagy (41–43). GPs are the main components of the cell membrane and play essential roles in cell proliferation, apoptosis, and differentiation. Abnormal glycerophospholipid metabolism causes changes in cell membrane composition and permeability, leading to aberrant cellular autophagy (44). In summary, we speculate that exposure to high alkalinity causes metabolic dysregulation in the carp liver and plays an important role in autophagy in carp hepatocytes by disrupting the structure and function of cell membranes and mitochondria.

Oxidative stress in aquatic organisms can be caused by environmental stress (45). Studies have shown that saline‒alkali stress can induce oxidative stress in aquatic animals and that alkali stress can lead to reductions in the levels of antioxidant enzymes in the gills of crucian carp (1, 46, 47). Alkali stress affects the antioxidant enzyme system of organisms by increasing the amount of reactive oxygen radicals produced during osmoregulatory energy expenditure and accelerating metabolism (21). Aminoacyl-tRNAs are essential for protein synthesis, and oxidative stress induces protein mistranslation by damaging the editing site of aminoacyl-tRNA synthetase (48). Our results revealed significant reductions in the concentrations of creatine, uracil, and aspartate. Creatine is a nutritional supplement that prevents damage to mitochondrial structure and function and restores cellular differentiation under oxidative stress conditions (49). It has been found that oxidative stress in the body leads to a significant increase in the ROS level and that excess H_2_O_2_ promotes the conversion of aspartic acid to pyruvic acid, thereby alleviating oxidative stress (50). *Nrf1* and *Nrf2* are essential for the maintenance of redox homeostasis and coordination of cellular stress responses, and the transcription factor *Nrf1* is activated by partial proteasome inhibition, ER stress, oxidative stress and hypoxia (51, 52). The targets of FoxO include genes encoding intracellular and extracellular antioxidant proteins (53). In the present study, oxidative stress was found to occur in the liver upon alkali stress. However, the expression of *Nrf1* mRNA increased and that of *Keap1* mRNA decreased upon alkali stress, which is an adaptive response of common carp to protect against liver damage caused by oxidative stress. In this study, we found that oxidative stress activates autophagy, a dynamically balanced process that maintains intracellular homeostasis. If this balance is disrupted, cell and tissue damage ensues. The results described above were obtained with two fish per group in triplicate experiments. To increase the stability of the model and reduce the bias caused by individual differences, in future research, we intend to increase the number of fish per group to five.

## 5 Conclusion

Through transcriptomic and metabolomic approaches, 61 DEMs, 4008 DEGs, and 1015 significant DMs were identified in the liver of common carp under alkali stress. KEGG pathway analysis was performed, and the results were combined with the transcriptomic and metabolomic analysis results. High alkalinity activated the mTOR signalling pathway, FoxO signalling pathway, MAPK signalling pathway, and autophagy signalling pathway. Alkali stress significantly altered the abundances of the metabolites cortisol 21-sulfate, histidine, creatine, and uracil, activating the glycerophospholipid metabolism and pyrimidine metabolism pathways involved in the regulation of autophagy. The target gene of *ccr-miR-140-5p* was identified as *ULK2*. Further studies indicated that exposure to high alkalinity can cause autophagy, mitochondrial damage, and oxidative stress. Therefore, our findings revealed that high alkalinity leads to metabolic disorders, induces oxidative stress and triggers mitochondrial autophagy through the *miR-140-5p–ULK2* axis. This study revealed the molecular regulatory mechanism of high alkalinity, providing new ideas and a theoretical basis for overcoming the detrimental effects of alkali stress in fish.

## Data availability statement

The data presented in the study are deposited in the SRA repository, accession number PRJNA1128732.

## Ethics statement

The animal experiment was performed according to the Guidelines for the Feeding and Application of Laboratory Animals of Heilongjiang Fisheries Research Institute, Chinese Academy of Fishery Sciences and was approved by the Committee on the Ethics of Animal Experiments of Heilongjiang Fisheries Research Institute, Chinese Academy Fishery Sciences (20230728-003). The study was conducted in accordance with the local legislation and institutional requirements.

## Author contributions

XS: Formal analysis, Writing – original draft, Writing – review & editing. LG: Data curation, Methodology, Writing – original draft. HW: Methodology, Writing – original draft. TL: Data curation, Methodology, Writing – original draft. XC: Formal analysis, Writing – original draft. WL: Methodology, Writing – original draft. YL: Methodology, Writing – original draft. XDS: Methodology, Writing – original draft. JL: Formal analysis, Methodology, Writing – review & editing. XT: Formal analysis, Writing – original draft, Writing – review & editing. WX: Funding acquisition, Writing – original draft.
